# The complete chloroplast genome sequence of *Oryza eichingeri* (Poaceae)

**DOI:** 10.1080/23802359.2017.1357452

**Published:** 2017-07-28

**Authors:** Fang Liu, Yan Zhao, Dengjie Luo, Dengwei Hong, Rongbai Li

**Affiliations:** State Key Laboratory for Conservation and Utilization of Subtropical Agro-Bioresources, Agricultural College, Guangxi University, Nanning, China

**Keywords:** *Oryza eichingeri*, the genus *Oryza*, chloroplast genome

## Abstract

The complete chloroplast genome sequence of *Oryza eichingeri* (GenBank accession number: KX085496) was generated by de novo assembly with low-coverage whole-genome sequence data. The chloroplast genome is 134,821 bp in length and showed conserved typical chloroplast structure. The cpDNA contained four rRNA, 39 tRNA, and 79 unique protein-coding genes. Seventeen genes contain one intron, only *ycf3* contains two introns; *rps12* is trans-spliced, one of its exons is in the LSC region (5′_end) and the two reside in the IR regions (3′_end) separated. A pair of gene *ndhH,* due to the 5′ part of *ndhH* which overlaps the IR/SSC junctions, was two unique genes. The AT content of *O. eichingeri* cp genome is 61%. Phylogenomic analysis showed that *O. eichingeri* is closely related to *O. officinalis*. The complete cpDNA of *O. eichingeri* provides essential and important DNA molecular data for further phylogenetic and evolutionary analysis for the genus *Oryza*.

Rice genus (*Oryza* L.), belonging to the grass family (Poaceae), consists of more than 20 wild species and two cultivated species, which widely grow in the tropical and subtropical regions (Vaughan 1994; Khush 1997), acting as the staple food for over half of the world’s population. *Oryza* species have 10 genome types (A, B, C, E, F, G, BC, CD, HJ, and HK) (Nayar 1973; Aggarwal et al. 1997; Ge et al. 1999), represent an enormous gene pool for genetic improvement of rice cultivars.

At present, some complete cp genomes belonging to Rice genus have been available in NCBI GenBank (http://www.ncbi.nlm.nih.gov/genbank), while the *Oryza eichingeri* cp genome has not been reported. In this study, we determined the complete chloroplast DNA sequence of *O. eichingeri* (IRGC Acc. No. 101422), a wild rice, by using next-generation sequencing technology. We filtered and assembled the complete cp genome with CLC Genomics Workbench v3.6 software (CLC Genomics Workbench v3.6 2010). The complete cp genome sequence with gene annotations was submitted to the GenBank (GenBank accession number: KX085496). The genome consists of 134,821 bp containing a pair of inverted repeats (IRs) of 20,819 bp, which was separated by a large single-copy region and a small single-copy region of 80,853 bp and 12,330 bp, respectively. The high plant cp genomes are AT-rich (Raubeson et al. 2007; Gao et al. 2009; Yang et al. 2010), and the *O. eichingeri* cp genome has alike characteristic, AT content is 61%.

The *O. eichingeri* cpDNA encodes 112 unigenes, including 79 unique protein coding genes, 29 tRNA genes, four rRNA genes. 43.17%, 2.01%, and 6.80% of the genome sequence encode proteins, tRNAs, and rRNAs, respectively, whereas the remaining 48.02% are non-coding and filled with introns and intergenic spacers. The four rRNA genes are all located in the IR. Twenty-one tRNA genes are located in the single-copy region, whereas the others are located in the IR. Seventeen genes containing one intron, only *ycf3* contains two introns; *rps12* is trans-spliced, one of its exons is in the LSC region (5′_end) and the two reside in the IR regions (3′_end) separated. A pair of gene *ndhH*, due to the 5′ part of *ndhH* which overlaps the IR/SSC junctions, was two unique genes. Gene *matK* was located within the intron of *trnK-UUU*, and ycf68 was located within the intron of *trnI-GAU*. In *O. eichingeri* cp genome, the pairs of genes *atpB-atpE*, *psbC-psbD*, and *ndhC*-*ndhK* had 4-bp, 53-bp, and 10-bp overlapping regions, respectively.

The maximum likelihood (ML) phylogenomic analysis of the 14 complete cpDNA from the genus *Oryza* showed that *O. eichingeri* is closely related to *O. officinalis*, they are CC genome types (Figure 1). The complete cpDNA of *O. eichingeri* provides essential and important DNA molecular data for further phylogenetic and evolutionary analysis for the genus *Oryza*.

**Figure 1.**
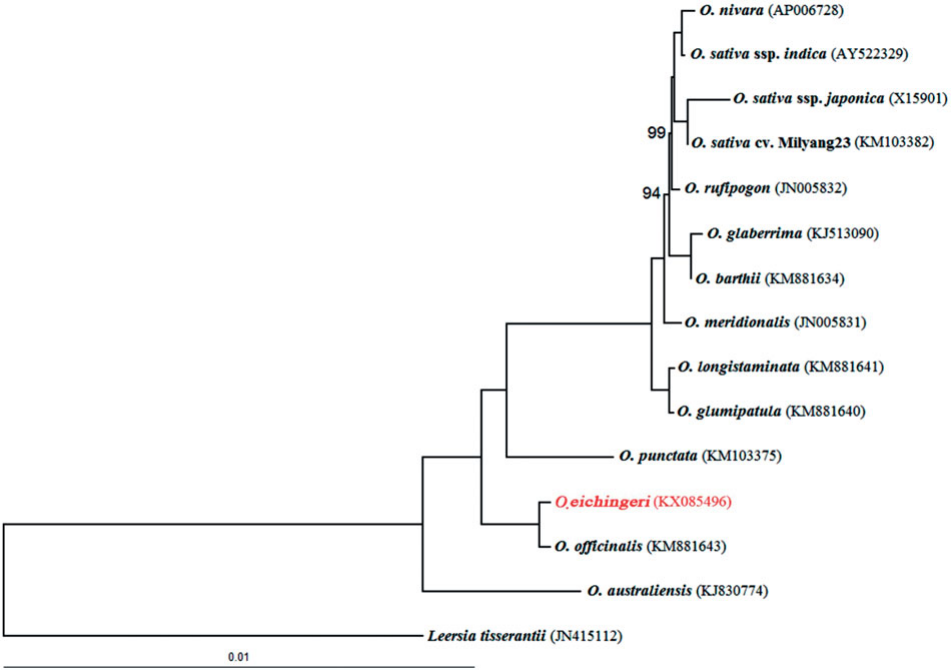
Maximum likelihood (ML) phylogeny of *Oryza* inferred from the whole-genome sequences of chloroplasts. Numbers near branches are bootstrap values of ML, the branches without numbers indicate 100% bootstrap supports. ML analyses were implemented in RAxML version 7.2.6 (Stamatakis 2006), GenBank accession numbers for sequences in brackets.
